# Supplementary material for: Microwave signal switching on a silicon photonic chip
Source: Sci Rep. 2019 Aug 1;9:11166. doi: 10.1038/s41598-019-47683-7 (PMC6672011; doi:10.1038/s41598-019-47683-7)
Supplement: Supplementary file 1 — Supplementary information [file 41598_2019_47683_MOESM1_ESM.docx]

**Supplementary information**

**Microwave signal switching on a silicon photonic chip**

**Cheng-Yi Fang,^1^ Hung-Hsi Lin,^1^ Mehdi Alouini,^3^ Yeshaiahu Fainman,^2^ Abdelkrim**

**El Amili^2*^**

1. University of California San Diego, Materials Science & Engineering Program, La Jolla, CA 92093
2. University of California San Diego, Department of Electrical & Computer Engineering, La Jolla, CA 92093
3. Institut FOTON, University of Rennes 1, CNRS, Campus de Beaulieu, Rennes, France
4. **Tapered- and through-type MORIMS design**


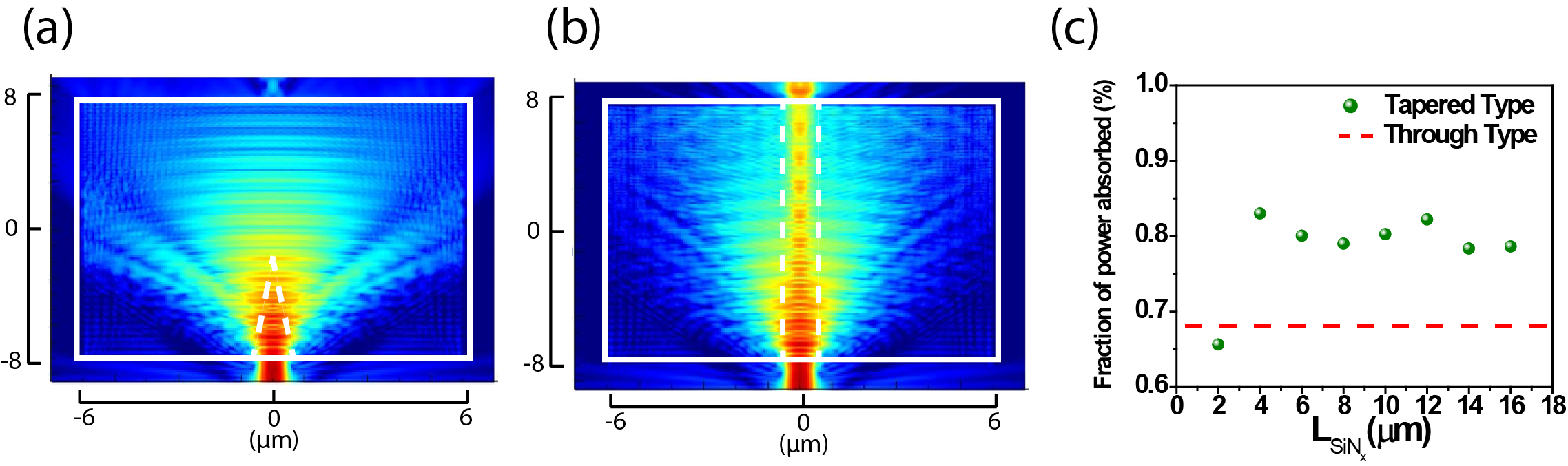


Fig. S1 Computed local electromagnetic filed |E| distributions at the height of 125nm of (a) tapered type structure and (b) through type structure in the Si photoconductive patch. The white solid lines indicate the Si photoconductive patch and the dash lines depict the SiNx waveguide on top of the Si photoconductive patch. (c) Simulated fraction of power absorbed by Si photoconductive patch of tapered type structure and through type structure with different SiNx taper length.

To optimize the transfer of the light from the SiNx waveguide to the Si patch, finite-difference time-domain (FDTD, Lumerical©) simulations were conducted. In the tapered-type design, the SiNx is tapered along the Si patch while the waveguide is crossing it completely. Figure S1a and b show how the amplitude of the optical electromagnetic field is distributed within the Si patches in tapered-and through-type respectively. With the tapered type structure, the incident light can spread out laterally within Si photoconductive patch. On the contrary, through type structure, the electric field spreads less and its energy is exchanged coherently between the SiNx waveguide and the Si patch along the propagation direction. This configuration allows the remaining light to be re-used for controlling another MORIMS nearby as shown later. Figure S1c shows the fraction of optical power absorbed by Si photoconductive patch with different taper length. The tapered-type structure allows ~84% of the light to be coupled into the Si photoconductive patch. The length of the taper is one of the main parameters that governs the coupling efficiency between the waveguide and Si slab. For sake of comparison, through type structures allow ~67% of the energy to be coupled into the silicon patch.

**II. Gap effect in Ground electrodes**


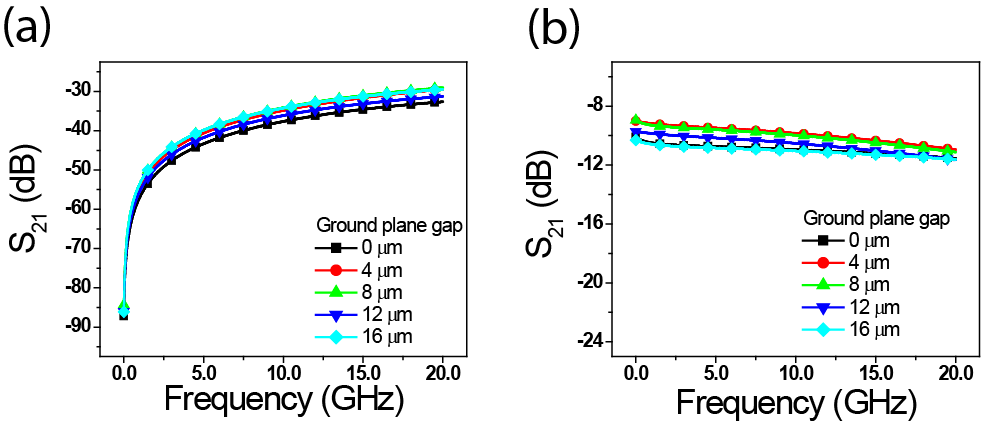


Fig. S2. Simulated S_21_ with different ground electrodes gap of (a) off state and (b) on state.

The ground-signal-ground metal contacts on both ends are designed to match the 50Ω impedance of the probes that connect to the microwave source. The specialty in our design is that the input and output SiNx waveguide cut through the ground plane leaving a gap between them. To investigate the effect of these gaps in the ground planes, we simulate the s-parameter with different ground plane gap using CST Microwave Studio. The device design is the same as shown in Fig .1 except the gap width in the ground plane. Fig. S2 shows the simulated S_21_ at both on and off states with different ground gap. At off state shown in Fig. S2a, where the intrinsic Si channel is not conductive, the S_21_ with different ground plane gap does not show significant difference. Fig. S2b shows the simulated S_21_ at on state (with Si conductivity of 2000S/m). Although the different ground gaps show slight different S_21_, compared to the 20dB on/off ratio, the ground plane gap does not affect the device performance. Moreover, the microwave wavelength is on the order of centimeter, so the micrometer scale ground plane gap does not play an important role in microwave signal transmission.

**III. Additional photoconductivity induced by the laser in Si patch calculation**

To further prove that the power consumption is small for MORIMSs, we therefore estimated the additional conductivity of Si photoconductive patch at on state through the following equation: ^1^

$\Delta\sigma_{s}=q(\mu_{n}{+\mu}_{p})a\beta\alpha\tau I_{p}$ (S1)

Where

$I_{p}=\frac{P\lambda}{Ahc}$ (S2)

Δσ_s_ is the additional conductivity under illumination, μ_n_ and μ_p_ are the mobility of electron and hole respectively, a is the term represents the amount of light sending into the Si photoconductive patch. β is the quantum efficiency. α is the absorption coefficient of Si at 800nm. τ is the carrier life time considering the surface recombination due to the small dimension of the Si patch. I_p_ is the photon density, P is the optical power, λ is the wavelength, A is the illumination area, h is the Plank constant and c is the speed of light.

The additional conductivity of the Si patch is estimated to be on the order of 2000 S/m when the incident power is at 2mW. Because our light absorbed Si region is only 16μm by 12μm, compare to the free space illumination scheme^2^, the compact device design has much lesser optical power consumption.

We then calculate the S_21_(on) at DC using the additional conductivity from Eqs. (S1)-(S2) through the following equation^3^:

$S_{21}\left( \mathrm{on} \right)=\frac{2Z_{0}Gg}{1+2Z_{0}Gg}$ (S3)

$Gg=\Delta\sigma\frac{A}{L}$ (S4)

Where Z_0_ is 50Ω, Gg is the conductance, A is the cross-section and L is the length of the Si photoconductive patch. The calculation shows S_21_ (on) is around -20dB when the additional conductivity is 2000S/m which is consistent to our experimental results at the incident power of 2mW. The S_21_ (On) of the device could be further improved by using doped wafer without sacrificing the R_on/off_.

**IV. An example of how MORIMSs can be applied in photonics**

We provide a schematic of an advanced architecture where the building blocks could for example route the light towards different MORIMSs or modulate the optical signal in order to introduce additional information/signal through mixing the RF/MW and the modulated optical signal.

The figure below is an example of simple schematic of how MORIMSs become part of an on-chip interconnect system. A directional coupler splits into 2 branches for controlling a phased array antenna for example. Each arm includes an optical Mach–Zehnder interferometer. Through controlling the phase difference in the MZI, we can, in addition, control the intensity of the optical power that will be fed in the MORIMs.


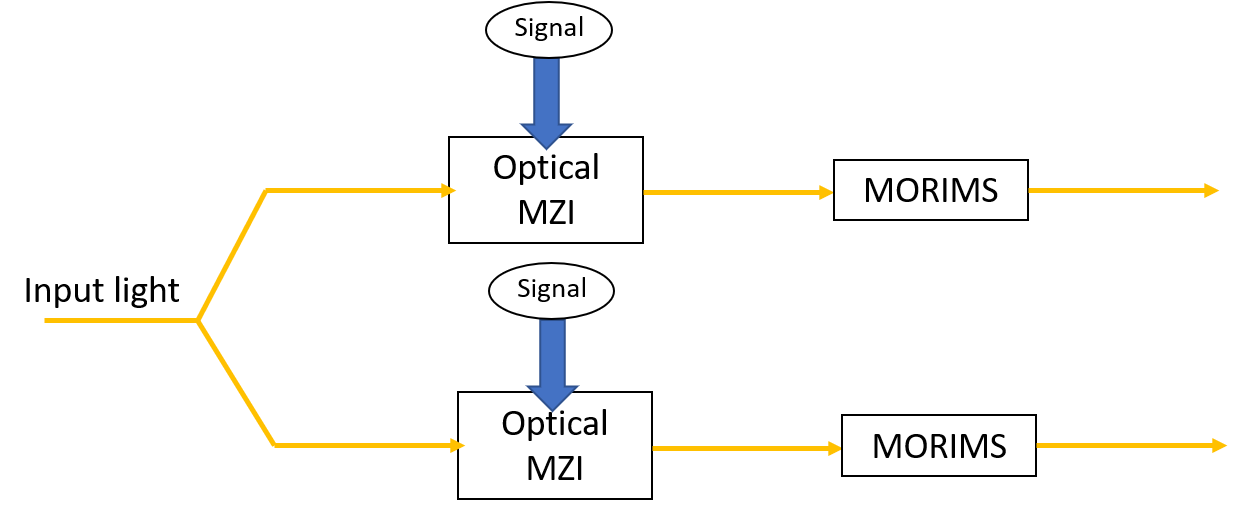


Fig. S3. Schematic of an advanced architecture where optical MZI can be combined with MORIMSs.

Moreover, the input light can indeed carry a signal, this can potentially be used as a mixer.

For sampling applications, it can also be done using such a scheme provided the time response of the photoconductive material is short enough. In this condition, we could control the optical MZI such that optical pulses with fixed interval in time domain feed the MORIMSs.

**Reference:**

1. Platte, W. Effective photoconductivity and plasma depth in optically quasi-CW controlled microwave switching devices. *IEE Proc. J - Optoelectron.* **135**, 251–254 (1988).

2. Lan, L., Zhao, D., Liang, F., Zhang, Q. & Wang, B.-Z. Influence of laser wavelength on insertion loss of silicon-based optically controlled microwave switch. *Microw. Opt. Technol. Lett.* **55**, 187–190

3. Tripon-Canseliet, C. *et al.* Optically controlled microwave phase shifting and sampling by efficient photoconductive switching on LT-GaAs substrate integrated technology. in *Photonics North 2006* **6343**, 63432K (International Society for Optics and Photonics, 2006).
